# Varieties and similarities of platform capitalisms: a comparative approach of labor regulation in Brazil, Portugal and Spain

**DOI:** 10.3389/fsoc.2025.1454324

**Published:** 2025-03-28

**Authors:** José Soeiro, Kenzo Soares Seto, Víctor Riesgo Gómez

**Affiliations:** ^1^Faculdade de Letras da Universidade do Porto, Instituto de Sociologia – UP, Porto, Portugal; ^2^Postgraduate Program in Communication and Culture, University of Rio de Janeiro, Rio de Janeiro, Brazil; ^3^Grupo de Estudios Críticos Urbanos (GECU), Universidad Nacional de Educación a Distancia (UNED), Madrid, Spain

**Keywords:** on-location platforms, platform capitalism, platform labor, uber, platform regulation, platform cooperativism, digital platforms, labor regulation

## Abstract

Digital platforms have led to the emergence of a new digital proletariat worldwide, subject to institutional arrangements that put the labor framework outside of labor law. However, this process of remercantilisation and informalisation of work has been accompanied by a transnational tug-of-war over the legal status of these workers. Using a comparative case study approach, this article seeks to describe and problematise the conflict surrounding the regulation of on-location platform work in Brazil, Portugal, and Spain. It looks at (i) the installation process and extension of the activity of on-location platforms in each country; (ii) the political, regulatory, and jurisprudential debate around the socio-legal framework of couriers and drivers, the role of intermediaries, and law-enforcement; (iii) the positions of the different collective actors and political agents (workers organizations, business lobbying, governments) on regulatory models; (iv) the alternative solutions put forward by movements and public policies, namely cooperative or ethically based platforms and their influence on the final model established in each country. The comparison between Brazil, Portugal, and Spain highlights how different national contexts – in terms of collective actors, labor and political institutions, and regulatory processes – regionally shape the global phenomenon of platform capitalism.

## 1 Introduction: platform capitalisms, labor transformations, and the importance of a comparative approach

We live in a world and a time strongly influenced by the emergence of new forms of value accumulation derived from the incorporation of information processing technologies into production. The notion of “platform capitalism” (Rahman and Thelen, 2019; Srnicek, 2016), allows us to direct our focus of this transformation to the growing influence of technological platforms as new socio-technical infrastructures in which is concentrated a large part of the production and accumulation of value in the global economies of the first quarter of the twenty-first century.

As Srnicek (2016) points out, one of the crucial points underlying the growing power of these infrastructures is their ability to extract and accumulate a vast amount of processed information in the form of data. Converted into a new type of capital (Sadowski, 2019), these data allows an infinite number of exchanges, including speculative ones, to be generated, increasing in value with each transaction. A specific imperative of platform capitalism consists of transferring as many activities as possible that previously took place offline to the platforms' own sphere of action, leading to a process of platformization of more and more activities (Sadowski, 2020; Srnicek, 2016). In its development, platformization has introduced significant transformations in the way work is provided and organized, having a profound impact on employment (ILO, 2024; Drahokoupil, 2021), redefining labor relations and challenging, differently in each country and region of the world, their regulations (Crouch, 2018).

The growing ability to mine and accumulate vast amounts of data has enabled platforms to attract large amounts of capital investment (Langley and Leyshon, 2017), offering investors the promise of becoming inescapable intermediaries in the economic sector in which they operate, forming quasi-monopolies that systematically elude the gaze of regulators (Khan, 2017).

Their infrastructural power comes from a “particular combination of socio-technical and commercial practices” (Langley and Leyshon, 2017, p. 13) that makes them owners of the playing field where interactions take place, thus gaining privileged control and the priority right of access to all the data produced by the different actors as a result of their interaction. Thus, they retain the ability to define those details that are considered sufficiently relevant to be taken into account and, consequently, to devote efforts to recording and storing them, which translates into regulatory power.

If we focus specifically on those platforms dedicated to providing services on the ground, which is the subject of this paper, we find, especially in the case of Uber, a use of the combination of its position as an intermediary and of the data collected that deserves to be analyzed.

On the one hand, Uber would be one of the pioneers in the process of dissolving the corporate structure as we have known it from the development of Fordism to the present day. For example, Rahman and Thelen (2019), following the approach of Davis (2016), have emphasized the fact that the data accumulated by Uber has allowed this company to articulate a new business management mode characterized by the apparent dissolution of the figure of the supervisor, limiting itself to algorithmically distributing the workload among an army of external workers who are considered as subcontracted partners, while at the same time this process served to weave new alliances between investors and consumers.

On the other hand, if we look at the way in which it entered the different markets where it launched its product, we see that one of the uses assigned to the data it accumulated was to develop different strategies to challenge the regulatory agents with which it was forced to deal, both in the case of the United States (Collier et al., 2018) and in the case of Europe (Thelen, 2018), acting as a company willing to engage in activities that at first glance appear to be loss-making in exchange for consolidating its positions (Shapiro, 2023). In this way, Uber gained a well-deserved reputation for being the leading exponent of a new wave of platform capitalism, characterized by defying all kinds of regulations in its attempt to expand. This seemed to foster the spread of a certain regulatory fatalism that could be expressed in the idea that platforms such as Uber could not be acted against.

This is also why, in certain circumstances, the emergence of platform capitalism seemed to herald the end of salaried work, labor law, the regulation of dismissals, the total liberalization of services and the expansion of liberalization and global competition (Degryse, 2016). There is no doubt that the platform economy has challenged existing mechanisms in the field of regulating freedom of association and collective bargaining, equal opportunities, occupational health and safety, pay, working hours, social protection and the termination of labor relations and the protection of personal data (ILO, 2024). But also, as Valdez (2023) shows, the truth is that, over time, digital on-location platforms have been forced to adapt their plans to the different regulatory contexts and strategies developed by the different political bodies with which they had to deal. In fact, many countries have already developed legal instruments, framework practices and regulatory interventions relating to the operation of platforms (ILO, 2024), as we will see in our case studies below.

The international debate on the regulation of platform work has been very intense, and there are multiple scales at which it has advanced, either at the instigation of local authorities, with the regulation of platform activities or the establishment of “ethical commitments,” or through case law, the approval of new laws aimed at specifically regulating the labor dimension of the platformised economy as a whole or more specific sectors (such as couriers or passenger transport drivers), or through collective bargaining (Barcevičius et al., 2021; Moreira, 2021; Van der Lann, 2023; ILO, 2024). This movement is also the result of struggles by workers in digital applications, which have been analyzed in the literature (Aslam and Woodcock, 2020; Chicchi and Marrone, 2024; Dufresne, 2021; Holgate, 2021; Moniz et al., 2021;) and the emergence of “platform unionism,” particularly in the European context (Aloisi et al., 2024).

The geopolitics of the platformization of work (Grohmann and Qiu, 2020) articulates global trends with the specific demographic, political, and social realities of each country, as well as its general insertion into the global economy and the international division of labor. Therefore, and taking these processes, their heterogeneity and contradictions into account, we believe it is imperative to discuss platform capitalisms in the plural, paying close attention to regional diversity (Steinberg and Punathambekar, 2022). That's why we propose, in this paper, a comparative analysis of three cases. Portugal, Spain, and Brazil are historically connected by the ties of the colonization process (having constituted a common state for half a century), by cultural similarities under the common Ibero-American framework, and by the relevant continuous migratory flows. Portugal and Spain are two countries of the Global North, but located in what we can consider “the southern periphery of Europe” (Bürgisser and Di Carlo, 2023). Brazil, is a dependent country on Latin America, historically on the capitalist periphery, but which has the ninth largest economy in the world ahead of the Iberian countries, occupying a regional leadership role and with ambitions of global relevance from the BRICS. The cultural ties between the three countries are reinforced by intense and continuous migratory processes between the two sides of the Atlantic. In turn, while Portugal and Spain share a semi-peripheral position with respect to the central countries of the EU, Brazil seems to enjoy greater regulatory sovereignty, being part of the BRICS group of countries, which contrasts with its position as a country of the so-called Global South.

We believe that a comparative approach that takes into account the regional dimension is useful to help focus the ongoing global debate on digital platforms and their influence on regulatory frameworks and approaches, as it is important to deepen the relationship between globalized processes and specific territorial conditions (Foster, 2024). By providing an original contribution focused on comparative analysis of Portugal, Spain, and Brazil, this papercloses a gap in literature, comparing the evolution of the phenomenon in countries that are culturally, geographically, and historically united, while at the same time situated in contradictory positions in geopolitical terms. This comparative approach is of particular interest in order to try to locate certain factors that may influence the stabilization of platform capitalism in each regional or local version.

First, we explain the processes of establishing the main platforms in each of the selected countries. We then map the social conflicts and the responses of the different actors in those processes. Thirdly, we analyze the presence or absence of cooperative alternatives and their influence on the final model established in each country. In the discussion section, we return to the most important aspects around which to draw comparisons and the results of the observation, focusing on the current situation in each of the selected locations. In doing so, we aim to highlight the unique and common characteristics of each national setting, offering an in-depth view of how different institutional and economic realities and traditions of workers' struggles influence the functioning of digital platforms, their regulation and the implications for workers.

## 2 Materials and methods: the comparative case study approach

We adopt a comparative case study approach (Bartlett and Vavrus, 2017) attending both to global trends and national variations. Case studies are based in three units of analysis: (1) the processes of establishing the main platforms in each of the selected countries, focusing on the regulatory gray zones that are exploited by the platforms for this purpose and the structural circumstances that facilitate it in each place; (2) the social conflicts that arise, and the responses of the different actors affected by the establishment process; (3) the presence or absence of possible cooperative or ethically based alternatives and their influence on the final model established in each country. To develop our comparative approach we chose relevant variables: the relative proportion of work on on-location platforms in each country; the sociographic characteristics of the working population in these sectors; the regulatory and socio-legal framework in each country; the position of platforms, trade unions, workers, and political parties on the regulatory framework; the identification of alternative platforms based on the cooperative or associative model. Our methodology has points in common with others used to conduct comparative studies on platform work between countries. For example, the Fairwork project, promoted under the auspices of the Oxford Internet Institute, carries out international comparisons based on the consideration of three complementary methodological strategies: documentary research, interviews with local managers of each platform, and interviews with platform workers in each place (Fairwork, n.d.). Our method of obtaining empirical material has replicated two of the three points of the Fairwork project (documentary research and interviews with workers).

Each of the authors conducted detailed research of each national case study, collecting qualitative and quantitative data, exploiting secondary sources such as national statistics from official bodies, official reports, legislation, judicial decisions, press releases, and public statements from the different actors, both the companies themselves and the workers' collectives. Field research was carried out in each country, including interviews with workers and participant observation at different events such as workers' assemblies, public demonstrations, strikes, and other protest actions. We developed a process-oriented and interpretative comparative approach from this data. The primary material from each site has guided a collaborative process of deliberation and discussion between the three authors to identify the most relevant aspects of practice and policy and contrast them with the situation in the other countries under analysis. The materials produced by each of us in our respective countries are pooled and the conclusions and observations are submitted for discussion, focusing on the aspects detailed throughout the paper.

## 3 The reality of on-location platforms in Brazil, Portugal, and Spain

The World Bank estimates the existence between 154 million and 435 million active workers on online platforms and the available data indicate a substantial growth in the number of active digital platforms, rising from 193 in 2010 to 1070 in 2023 (ILO, 2024, pp. 15, 18). Most of these platforms operate in the delivery sector (334) and in individual transport of passengers (119) and the majority of workers on platforms are engaged precisely in these two sectors: delivery and passenger transport; in 17 countries in Europe, around 33.3% of platform workers are engaged in the delivery sector and around 13% for passenger transport. (ILO, 2024, p. 20). Given the significant concentration of platform workers in the delivery and ride-hailing sectors, the following section provides an overview of their weight, sociographic composition and working status (Table 1).

**Table 1:** Main characteristics of platform work in each country.

**Aspects**	**Brazil**	**Portugal**	**Spain**
Number of platform workers	703,280 drivers and 588,550 couriers (regular workers). Around 1,7% of the labor force	69,791 drivers; estimated thousands of couriers. Around 1,4% of the labor force	Estimated 15,000–20,000 ride-hailing drivers; 12,000 couriers with contracts. Around 1% of the labor force
Main platforms in each country	iFood (dominates delivery market), Uber (dominates ride-hailing), 99, Rappi	Uber, Bolt (ride-hailing); UberEats, Glovo (delivery)	Cabify, Uber, Bolt (ride-hailing); Glovo, UberEats (delivery)
Presence of national platforms	Strong presence (e.g., iFood) in delivery market	Almost total dominance of foreign platforms	Relevant national platforms in delivery market (Glovo) and ride-hailing (Cabify)
Influence of the informal economy	High structural informality	Low informality in transport sector, but precarious work; informality in courier	Less informality than Brazil, but still precarious
Impact of immigration on platform work	Low presence of immigrant workers	High presence of immigrant workers, especially in delivery sector	High presence of immigrant workers
Position in the global economy	Regional leader in Latin America	Peripheral position in the EU, no regional leadership	Peripheral position in the EU, regional influence over Portugal

Brazil has 1.49 million regular platform workers according to official data (IBGE, 2022), which represents 1.7% of its economically active population, although other research estimates that 11.4 million Brazilians use apps as a complement or main source of income (Fioravanti et al., 2023). In total, 47.2% of these workers work in private transport via apps and 39.5% in delivery via delivery apps, while 13.9% use taxi apps and 13.2% general service platforms, such as microtasks (IBGE, 2022).

The transport and delivery app labor markets are highly monopolized in the country. Uber is used by all app drivers in Brazil, while 98% of delivery workers use the Brazilian platform iFood. However, many workers use more than one platform, with 32% of drivers using China's 99 and 9% of delivery workers using Colombia's Rappi (Datafolha, 2023).

This concentration reflects consumer preference. Since 2014, Uber has reached 500 cities in Brazil, serving 30 million passengers with one million drivers (Uber, 2024). iFood, founded in 2011, is the leader in delivery in Latin America, controlling 80% of the Brazilian market, with 43 million consumers, 250,000 regular delivery drivers and 870,000 workers connected to the platform to some degree (iFood, 2024). Its dominance even resulted in UberEats, Uber's delivery platform, leaving Brazil, alleging monopolistic practices by iFood (Seto, 2023).

Platform workers in Brazil work an average of 45 hours a week (Camelo et al., 2022), with monthly pay ranging from $360.00 to $630.00, and 60% of them do not have access to any social security (IBGE, 2022). Although the income of platform workers is 5.4% higher on average than that of non-platform workers in the country, there are major regional discrepancies. In the Northeast, the poorest region, the average income of platform workers is 23.5% higher than the others, while in the Southeast, the richest region, platform workers earn less on average than the others (IBGE, 2022). Finally, it should also be noted that these workers are mostly male, up to 40 years old and black or brown (Festi et al., 2024), demonstrating a significant gender and racial divide.

Portugal, for its part, has the third largest workforce performing platform work out of 14 European countries (Urzi Brancati et al., 2019; Boavida and Moniz, 2022). According to a study carried out in 2017, more than 10% of the total adult population had then provided some service from a digital platform, with between 2% and 4% of workers having digital platforms as their main (or only) source of income (Pesole et al., 2018). It is impossible to know for sure how many people are working on these on-location or “offline crowdwork” platforms. In the case of passenger transport using digital platforms, the number of companies and licenses is known there are 16.814 “operators” and 69.791 drivers, spread across four main nationalities: 42.014 Portuguese, 13.085 Brazilians, 6.352 Indians, and 2.182 Pakistanis (IMT, 2024: 18) (Instituto da Mobilidade e dos Transportes, 2024, p. 18). In the case of couriers, the lack of a specific law and the prevalence of informality make the task of gauging the number of workers more difficult, although it is probably in the tens of thousands. The law enforcement agency identified 93% foreign workers among the couriers, mainly Brazilians (60%) and Hindustanis (more than 30%) [Autoridade para as Condições de Trabalho (ACT), 2024, p. 25].

In Portugal, the passenger transport and food delivery sector also show the international trend of a higher percentage of migrants from third countries than in other platformized sectors (namely cloud work) and then in non-platformized work in general (cf. Zwysen and Piasna, 2024, pp. 12, 16). In the case of Portugal, the presence of workers from Brazil and the Indian subcontinent is particularly noticeable, the greater prevalence of which is related to the regulatory obstacles they encounter and the greater discrimination in accessing the formal labor market identified in studies in other countries (Zwysen and Piasna, 2024, p. 27; Fonseca and Soeiro, 2024).

Passenger transport via digital platforms began in Portugal in July 2014 with Uber. Currently, the two largest operators are Uber and Bolt, but there are eight registered platforms. Delivery platforms have been operating in Portugal since 2017, the largest being UberEats (with 30% of the market) and the Spanish Glovo (with 20%), joined by others such as BoltFood or TakeAway.com (Boavida et al., 2021, p. 14). There is only one fully Portuguese delivery platform that has had a regional presence, Xico's, albeit very localized. The business operates as described above, with payments being made, as a rule, through a fixed fee per delivery, a variable fee depending on kilometers and a percentage of the amount charged for the delivery, which is retained by the platform (usually 25%). The favorable institutional environment for these companies (particularly from the point of view of legal frameworks, but also a cheap and relatively qualified workforce); and the lack of case law, until the end of 2024, that has determined the attribution of employment responsibilities to digital platforms, unlike what happened in other European countries, have been factors that make the country attractive for the development of this type of platform capitalism.

In the case of Spain, it was in 2013 that an application aimed at offering private passenger transport services, provided with high-end vehicles and professional drivers, was promoted to the public by the Spanish company Cabify. Its founder had raised funding by selling a product that aspired to be the “Uber of Europe” (TechCrunch, 2012). A year later, another local company, Glovo, was founded, in this case with the intention of operating in a sector that was new at the time, namely the delivery of food by app. The emergence of platform work on Spanish soil was thus initially driven by two companies of Spanish origin.

At present, the real impact of platform capitalism in the home-delivery and private transport sectors in Spain is difficult to quantify precisely. On the one hand, there are no official figures available. It is only possible to make estimates based on other data. Specifically, in private transport, in January 2023 the number of authorizations in circulation stood at almost 18,000 VTC (Spanish acronym for Tourist Vehicle with Driver) licenses spread throughout Spain [Ministerio De Transportes, Movilidad y Agenda Urbana (MTMAU), 2024]. However, it must be considered that one of the limits they have is that the activity must be restricted to the regional area in which the authorization is issued. On the other hand, the technology platforms Uber, Cabify, and Bolt, the only ones present in the Spanish market, only offer their services in some of the big cities, mainly Madrid, Barcelona, Valencia, Bilbao, Zaragoza, Seville, and Malaga. Considering that a significant number of licenses are operated on double shifts, we could be talking about a figure of between 15,000 and 20,000 platform workers in this sector spread throughout Spain, although with a much higher incidence in the metropolitan area of Madrid, which concentrates almost half of the licenses in the whole country.

It is even more complicated to know the number of people working in the delivery sector. A report published under the auspices of Esade, a private academic institution with close links to the business world, estimated 12,000 workers with permanent contracts in the delivery sector (Esade, 2022). However, this could be just the tip of the iceberg, as there are frequent reports of sanctions initiated by the labor inspectorate against platforms for failing to comply with the hiring obligation set out in the Riders' Law (Olías, 2024). Migrants are more likely to engage in platform work than native-born: 10% of share of platform workers among the migrant populations of working age in Spain, compared with 5% of native working population (Zwysen and Piasna, 2024, p. 18),

Considering this first picture, it is already possible to highlight the weight of platform work in Brazil and Portugal, which seems to be lower in Spain. The Spanish economy seems to be less exposed to platform capitalism than the other two. Perhaps this can be explained by the larger aggregate size of the Spanish economy compared to the Portuguese one, as well as a lower weight of informal labor in the Spanish case compared to Brazil. On the other hand, the reality of locally sourced platforms is very important in Spain for drivers and delivery, and in Brazil for delivery, while in practice it is non-existent in Portugal. In any case, one of the aspects that makes comparisons difficult is the lack of comprehensive surveys in Portugal and Spain, especially by official bodies, either through public statistical agencies and their survey instruments, or by using company data, which are not provided to the states.

## 4 Troubled installation processes: the socio-legal framework of couriers and drivers and the political, regulatory, and jurisprudential conflicts in Brazil, Portugal, and Spain

Platform work and the use of algorithms to organize, supervise and evaluate work poses very relevant challenges to labor regulation. These include the definition of the employment status of the workers, the definition of their remuneration and working time, the access to social security and occupational safety and health, their collective voice, the rules of the termination/deactivation and the way algorithms affect working conditions and are accountable and transparent (ILO, 2024). Different countries have been regulating platform work in a variety of ways, and that's the case of Brazil, Portugal, and Spain (Table 2).

**Table 2:** Regulation of platform work in each country.

	**Brazil**	**Portugal**	**Spain**
Regulation status	Intense legislative debate, no consolidated regulation	Drivers: no employment relationship with the platform, but possible with intermediary. Riders: presumption of employment in the new labor law	Drivers: standard labor contracts and VTC licensing system. Riders: Law recognizing employment relationships for delivery workers
Main labor laws	Law 13,640/2018 (regulates app-based transport), Proposal PL 12/24 (under debate)	Law 45/2018 (“Uber Law” for transport); Law 12/2023 (“Agenda for Decent Work”)	Riders' Law (2021) for delivery workers, stricter ride-hailing rules under VTC framework
Judicialization of labor relations	High number of lawsuits, conflicting court rulings, Supreme Court rejecting employment relationship	No lawsuits in transport sector due to the TVDE operator framework. Diversity of decisions on riders' employment status, due to the new law	Many lawsuits leading to regulation

In Brazil, the platformization of work emerged in a general context of flexibilization of labor legislation, with the Temer government's labor reform following the impeachment that ended the Workers' Party's 14-year rule (Seto, 2021). Temer's reform was the biggest change to the Consolidation of Labor Laws (CLT), Brazil's main labor regulatory framework since 1943. The new law included extending the working day from 8 to 12 hours a day, the primacy of collective agreements over labor legislation, among other measures to make work more precarious with the justification of increasing the supply of vacancies after the 2008 crisis (Seto, 2021).

One of the innovations of this legislative intervention was the creation of the category of intermittent work, characterized by the provision of non-continuous services for indefinite periods, in which workers have fewer rights, such as the absence of unemployment insurance (Seto, 2021). It was by reference to this recent legal figure that the Brazilian labor courts began to recognize, in some cases, the employment relationship between professionals and platforms, considering that these manifest “direction of services,” a characteristic of an employer relationship according to the CLT, even if remotely, thus applying the new category of intermittent work (Oliveira et al., 2020).

In 2018, the government also regulated individual transport by app through law 13,640, mentioning platform work for the first time. Prior to this, platforms such as Uber operated in an uncertain legal environment, as previous legislation granted a monopoly on paid passenger transport to taxi drivers, with more than half of Brazil's capitals seeing protests by taxi drivers proposing bills to ban Uber by 2015 (Barifouse, 2015), as happened in other countries including Spain and Portugal. Although the 2018 Brazilian law does not establish an employment relationship between professionals and platforms, it does set minimum requirements for drivers to work, including a specific driving license for paid activity, no criminal record and social security contributions.

Subsequent governments have proposed a new category for platform workers, differentiated from the status of salaried workers, which would guarantee a limited set of rights compared to the others. The Bolsonaro government announced a provisional measure in 2022 to regulate platform labor, but it has not been implemented (Doca, 2022). In the Brazilian Congress, several bills have been proposed, such as PL-1471/2022, seeking to establish minimum wages for workers, but none have been approved.

At the same time, more than 10,000 lawsuits on labor relations between workers and platforms have been filed in the labor courts, with conflicting decisions [Supremo Tribunal Federal (STF), 2023]. In 2023, Brazil's Superior Labor Court (TST), the highest court of labor justice, recognized the labor relationship between workers and platforms, but Uber appealed to the Federal Supreme Court (STF). The STF has renounced the application of labor law between professionals and platforms, considering platform work to be valid outsourcing [Supremo Tribunal Federal (STF), 2023]. In 2024, the STF should consolidate this understanding in a general repercussion judgment, definitively denying the validity of current labor legislation for platform work [Supremo Tribunal Federal (STF), 2023].

Against this backdrop, in 2023 the Lula government set up a “Grupo de Trabalho Tripartite” (GTT) with representatives from the executive branch, the workers and the platforms, to draw up a specific law for platform work. Faced with the lack of consensus within the Group, in 2024 the government sent the Complementary Bill 12/24 (PL12/24) to Congress, which only regulates the work of drivers. This bill sets limits of 12 hours a day per platform, without exclusive dedication, minimum hourly pay and social security, with the social security contribution divided between workers and platforms. From the point of view of labor relations, guidelines are established for the suspension of workers by the platforms, guaranteeing the right to defense, greater transparency and access to data on workers is mentioned and the creation of state-recognized unions for this category is made official.

In Portugal, despite the similarity in an initial public dispute over the very legality of Uber's operation, the situation is quite different from the one in Brazil. As has been said, the installation of digital platforms in Portugal took place in 2014, in the case of passenger transport, and in 2017, in the case of delivery. We can place the first phase of the process of platformization of work in Portugal between 2014 (the year the activity began) and 2018, the year the first law was passed to specifically regulate the “activity of individual and remunerated passenger transport in uncharacterized vehicles from an electronic platform” (Law no. 45/2018). It was also during this period that the growth in the presence of foreign workers living in Portugal accelerated, becoming increasingly diversified, a relatively new reality in Portugal and with great expression in the platform transport and delivery sector. It is at this stage, as Tomassoni and Pirina (2022, p. 249) point out, that Portugal, and Lisbon in particular, becomes “the laboratory for the expansion of a platform capitalism that is very much supported by the public authorities,” and it should be noted that Uber chose the Portuguese capital to set up its technology center in Europe.

Law 45/2018, known as the “Lei Uber,” which regulates passenger transport, is a specific law that has enshrined the existence of three mandatory legal elements (rather than two, as in most countries) in the operation: (i) digital platforms, (ii) TVDE (Transport in Unmarked Vehicles from Electronic Platforms) operators, and (iii) drivers. The digital platforms must comply with regulations that determine the information made available and presented to the consumer of the service and are charged an intermediation fee of up to 25%. TVDE operators must be a licensed company in Portugal and are responsible for recruiting drivers, owning the vehicles and providing the transport service. Drivers may have a contractual relationship (subordinate labor or self-employment) with the operators, but, according to the law, not with the platform. As well as contributing to the liberalization of the private transport service (Guerreiro and Marques, 2023), this law was considered particularly “original” for introducing the mandatory figure of the “TVDE operator” which had the effect (and possibly the purpose) of making less clear the contractual link between the driver and Uber or another company operating the platform (Amado and Moreira, 2021), thus legally freeing digital platforms from labor commitments. In fact, although the law places a number of typical employer powers in the legal sphere of the platforms, such as the control of working time (Moreira, 2021, p. 95), it does not stipulate that there can be any employment relationship between workers and platforms. If there is any employment relationship, it will, according to this law, be between the worker and the “TVDE operator,” and it remains to be seen how many of these operators are not, in fact, corporatized workers, i.e., false entrepreneurs (Soeiro, 2024a,b). It's no wonder, then, that at this stage Portugal hasn't had any court cases recognizing employment contracts between passenger transport platforms and workers.

A second phase, between 2019 and 2023, took place when a broader debate began on the regulation of digital platforms, which was expressed in the preparatory work and then in the publication of the Green Paper on the Future of Work (Moreira and Dray, 2022), launched in 2021 by the Portuguese government, and culminated in the approval, in February 2023, of the so-called “Agenda do Trabalho Digno” (Law 12/2023), which includes a set of changes to labor legislation, namely a new “presumption of employment contract in the context of a digital platform,” which came into force in May 2023 (see Amado, 2023). This was the phase of the great expansion of platforms, particularly during the pandemic period, generating a series of debates on “essential workers” and their protection and on the digitalization of work, particularly due to lockdowns and the shift to teleworking. In line with international jurisprudence, the Green Paper on the Future of Work assumed the relevance of “creating a presumption of labor adapted to work on digital platforms, to make the distinction between employees and self-employed workers clearer and more effective.”

This reality was enshrined in the new general labor law, in force since May 2023, which provides for this presumption of the existence of an employment contract between the activity provider and the digital platform when some (at least two) of the characteristics listed in six paragraphs are met, namely: fixing the remuneration, exercising management power, exercising control and supervision over the provision of the activity, restricting the activity provider's autonomy regarding the organization of work, exercising disciplinary power and the existence of work equipment and instruments belonging to the digital platform (Law 13/2023; Amado, 2023).

A third phase has begun since then and concerns the application of the new labor law and the legal dispute over the labor framework of this activity. This phase has been characterized by intense inspection activity, with 1217 notices issued by the Working Conditions Authority regarding work on digital platforms, giving rise to 884 reports to the Public Prosecutor's Office for recognition of employment contracts with the platforms, the majority of which concern couriers and only 14 drivers of uncharacterized passenger transport via digital platforms [Autoridade para as Condições de Trabalho (ACT), 2024; Diário da República, 2024]. It has also been the phase in which various judgments have begun to be handed down, in different directions. The majority of first instance decisions have not been in favor of recognizing the employment contract, as the Public Prosecutor's Office claimed, but appeal decisions from higher courts have been in favor in 80% of cases−17 out of 14 (Martins, 2025). In this period there has also been an intensification of protest movements by couriers and drivers, with several stoppages in the main Portuguese cities, called by informal groups and industry associations.

In the Spanish case, it is necessary to situate the emergence of digital platforms in a context of a labor market in which the oscillations in the unemployment rate are much more marked, both in times of economic boom and in recessionary phases, than in the rest of the European Union countries (Ayala and Cantó, 2020). In Spain, economic recessions immediately translate into job destruction, in an institutional context governed by high de facto labor flexibility (Martínez Pastor, 2022) and high rates of temporary employment. The global recessionary cycle that began in 2008 was deepened by economic policies aimed at structural adjustment, although its results can be described as “austericide” (Gálvez Muñoz, 2021). Unemployment rate soared to around 25% during 2012, 2013, and 2014, combined with substantial increases in inequality (Ayala and Cantó, 2020). It is in this context that different technological work platforms appeared in Spain. In the private transport sector, Uber launched its services in 2014, the same year as in Portugal, although the local platform Cabify had already been offering private rides through a technology platform for at least a year, targeting an exclusive segment of the market (Riesgo Gómez, 2023c). Almost simultaneously, in 2014, the food delivery platform Glovo was founded in Barcelona, extending its service offering to the rest of Spain, entering into competition with Deliveroo, a British platform that was introduced in Spain in 2015 (Fernández-Trujillo Moares and Gil García, 2021).

The responses to these launches differed depending on the economic sector we are looking at. Although all the platforms initially tried to frame their service offerings within the framework of the so-called “sharing economy” (Botsman and Rogers, 2010), there are profound differences between the business model of Uber or Cabify, which competed directly with a highly regulated sector in Spain such as the taxi sector (de-Miguel-Molina and Catalá-Pérez, 2021), and the business model of food delivery, in which there were no companies operating beforehand. In fact, Uber's peer-to-peer service was pursued and banned within a short period of time. By the end of 2014, it was no longer possible to hire a vehicle driven by a private individual through the Californian platform (Riesgo Gómez, 2023c). Commercial courts in Madrid and Barcelona took the legal decision at the behest of taxi drivers' associations that denounced the provision of this type of service. However, food delivery platforms managed to establish their business model without raising too much controversy in the initial stages. In the context of high unemployment, their appearance made it possible to provide income to a part of the population whose access to the labor market was completely closed, although this meant accepting the platforms' refusal to assume the obligations derived from an employment relationship in a clear escape from labor law.

Shortly after its ban, in March 2016, Uber returned to the Spanish market, adopting in this case a legal strategy to be able to operate. Together with Cabify, both platforms began to operate a particular type of private transport license, the Vehicle Transport with Driver (VTC) authorizations, initially conceived for the provision of high-end personalized services. This type of administrative permit was subject to strong legal restrictions on aspects such as quantitative limits and modalities of services that could be provided. However, heterogeneous groups of local investors, sometimes financially backed by the platforms themselves (Riesgo Gómez, 2023c), pursued a strategy of judicial confrontation, filing lawsuits in administrative courts that bore fruit, thus succeeding in expanding the number of VTC authorizations initially foreseen in the law. In this way, the lack of clarity in the legislation was exploited, forcing different legal interpretations on some of the essential aspects related to the regulator's original intention (Guillen, 2018). The result of all these interpretative controversies influenced the business structure present in the sector, which became dominated by a small group of investors who controlled most of the authorizations, closely linked to the platforms themselves (Riesgo Gómez, 2023c). This meant, in turn, the almost total implementation of a labor model characterized by the majority of drivers in the sector being hired under the general regime as salaried workers, albeit with such particular working conditions that they can be considered as “false salaried workers” (Riesgo Gómez, 2023b). Only because of the trade union presence has it been possible to gradually improve these conditions, bringing them, in some cases, closer to those of a standard employment relationship model (Riesgo Gómez, 2023a).

For their part, the absence of an employment relationship that the platforms tried to impose in the delivery sector took the form of the obligation to register as a self-employed worker for those who were recruited to work with them (Soto, 2023). This model began to explode thanks to the complaints filed throughout Spain in 2017. From the first rulings, one issue was becoming clear: the labor model under which the riders had been working until then was operating in fraud of the law, and they could be considered bogus self-employed. In order to regularize the situation, according to these rulings, it was necessary for the platforms to take on the employment of their delivery staff. However, this was not definitively consolidated until 2020, when the Supreme Court issued the first ruling in this regard, closing any possibility of appeals to other judicial bodies by the platforms.

As we have seen, in each of the countries digital platforms have exploited different legal frameworks, taking advantage of loopholes in the regulation of activities or manipulating contractual modalities envisaged for other realities and appropriated by the platforms to develop their business model. This highlights the different national strategies and also the differences between the transport and delivery sectors.

In Brazil and Spain legal action has been the first resort for platform workers to challenge platforms' classification of their employment status and the terms and conditions of their service contracts (ILO, 2024), which couldn't happen in Portugal because of the specific law on transportation via digital platform. In Spain and Portugal, the process ended up with new provisions (in a specific law or in the general law), aimed at addressing the issue of the classification of employment relationships with platforms. Intermediaries are specifically referred in the Portuguese law, and they also operate via outsourcing in Spain and Brazil.

## 5 Social struggles, trade union intervention, business lobbying, and government positioning in the platform ecosystem

An analysis of the regulatory differences and the similarities and specificities of platform capitalisms in the three countries cannot be done without considering the role and positions of the main players in these processes: workers, the organized labor movement, companies, and their representatives and the state, particularly the government (Table 3). It is between these agents that the main disputes over changes to the socio-legal framework for platform activity and the recognition of workers' rights have taken place.

**Table 3:** Organization of workers, lobbying of platforms and political disputes over regulation.

	**Brazil**	**Portugal**	**Spain**
Main worker organizations	ANEA (National Alliance of Delivery Workers), AMASP (São Paulo Association of App Drivers)	National TVDE Association; Estafetas em Luta; Associação de Imigrantes e Trabalhadores por Aplicação	Riders por Derechos, traditional unions like UGT and CCOO also involved
Relationship between traditional unions and platform workers	Distant relationship due to labor precarization	Weak relationship and declining unionization	Strong collaboration between unions and worker collectives
Main demands of workers	Focus on minimum pay and better working conditions	Focus on immediate economic improvements	Focus on employment recognition and algorithmic transparency
Main strategies of platforms	Political lobbying, attempts at self-regulation	Changes in operation to detour the law; use of employer associations to shape public opinion	Strong resistance to regulation, corporate lobbying
Experience with platform cooperatives	Experiments with cooperatives and public platforms (e.g., TaxiRio, AppJusto, Señoritas Courier)	No significant experience with cooperatives	Experiences with cooperatives (e.g., La Pájara, Mensakas)

In Brazil, despite exploring legal figures around intermittency, the platforms began by claiming that the lack of personal hierarchy and fixed working hours characterized the autonomy of the professionals who use them, opposing the recognition of any employment relationship (Antunes and Filgueiras, 2020). However, a fundamental political event transformed the Brazilian platform labor scene: the “*Breque dos Apps*,” the first national strike by delivery workers in Brazil in 2020, which managed to affect the operation of platforms in almost every state (Souza, 2023). The strike was the result of the confluence of regional mobilizations from newly founded delivery associations with a local reach and small national networks of activists, and sought better working conditions, but without a unified agenda on labor regulation (Souza, 2023).

In response, iFood ran anonymous paid ad campaigns to promote discourses such as “without a boss and minimum wage, delivery workers have more freedom and earnings” among workers (Rup, 2022, p. 1). However, following legal action, iFood signed an agreement with the Labor Prosecutor's Office that prohibits negative advertising by the company about labor rights (Castro, 2023).

Since then, there has been a transformation in the attitude of the platforms, with Uber, iFood, 99, and Amazon joining other platforms in the Brazilian Mobility and Technology Association (Amobitec), which since 2023 has officially supported the regulation of platform labor (iFood, 2024). iFood, in particular, promoted the National Delivery Workers Forum, bringing together delivery workers who are YouTubers with great influence in the community and local leaders from all over the country, as well as promoting “voice of drivers” meetings with their workers in each region of the country (iFood, 2024).

In the debate on regulation, it is also worth mentioning the importance of academic researchers, especially the Fairwork (2023) project, coordinated by the Oxford Internet Institute, with a Brazilian section, which seeks to define decent platform work based on parameters such as pay, safety, work management, and respect for workers' self-organization. Fairwork (2023) regularly monitors platforms and is institutionally recognized by the Ministry of Labor and by associations of platform workers.

With greater organization and production of data, there is a dispute over the model for regulating platform work. According to the available surveys, most drivers and delivery workers reject the employment relationship because they believe it limits their autonomy by imposing working hours and locations (Datafolha, 2023; Festi et al., 2024). However, 89% of workers support more rights with flexibility, with 75% saying they would contribute to social security if the deduction was automatic and shared with the platforms, as long as they retained the autonomy to choose services (Datafolha, 2023). This support varies enormously depending on the rate of contribution, with 66% of workers in favor of handing over 1% of their income to the state, but only 25% tolerating a social security contribution of 10% of their income (Datafolha, 2023). In the case of delivery drivers, most workers prioritize more immediate demands such as an end to double or triple rides, an end to blockades without defense and support points distributed throughout the city, as well as discounts and subsidies for the purchase of a work vehicle. From the point of view of social security, they are also in favor of sickness benefits. However, only around a third of those interviewed by Festi et al. (2024) prioritize the right to strike and minimum pay, and around 10% are in favor of an employment contract or a limit on working hours.

These controversies were expressed in the tripartite group (GTT) created by the government to formulate the platform labor regulation project. With workers' representation divided between traditional trade union centers and the new delivery and driver organizations, such as the *Aliança Nacional de Entregadores* (ANEA), as well as employers' organizations divided into two associations, there was no consensus and the GTT ended without a unified agenda.

However, President Lula, who had created the Group, sought global leadership on the issue, opening the UN General Assembly with a speech stating that “platforms should not abolish the labor laws we fought so hard for” (Da Silva, 2023, p. 1). At the same event, together with former US President Biden, he launched a “Global Coalition for Labor” pact between the US and Brazil to promote labor rights (Sanchez, 2023). Despite the impasse in the GTT, Lula also sent PL 12/24 to the Brazilian Congress, aimed exclusively at drivers, claiming that iFood would have been the biggest impediment to a unified proposal with delivery workers (Bandeira, 2024).

Despite the president's speech presenting this legislative proposal as a labor measure, the National Alliance of Delivery Workers (representing delivery workers) accused the government of favoring companies with its bill (iFood, 2024) and, in fact, Uber supported PL 12/24, while drivers and even delivery workers held several demonstrations against the platforms and the bill (Moncau, 2024; Brigatti, 2024). The protesters argued that the proposed payment floor could become an earnings ceiling, with the platforms limiting the number of rides for the driver after reaching the minimum amount (Moncau, 2024). There is also the claim that the bill only benefits the state—seeking to increase its revenue through the mandatory social security contribution, set at 7.5% of workers' income—and the traditional unions, which many workers do not consider representing them. For example, the Brazilian government's bill stipulates that working hours can only exceed 12 hours per platform if negotiated by the unions, which goes against associations such as the São Paulo Association of App Drivers (AMASP), which stated that “the unions will have total power over the class (…) however, the unions have no representation whatsoever in our class and we affirm that they do not represent us” (Brigatti, 2024, p. 1).

The majority of platform workers are not unionized, nor do they wish to be (Datafolha, 2023; Festi et al., 2024), and there also seems to be a broad rejection of political parties. The new forms of collective organization of platform workers repudiate the official union structure and highlight the relevance of platform ecosystems for their own self-organization, such as WhatsApp groups for calling strikes and delivery workers who become YouTubers and thus influential leaders among workers. The platformization of the workers' self-organization process implies a new political culture of mobilization and association that remains a challenge to incorporate into traditional trade union work. The crisis in the representativeness of the official unions was one of the elements in the platform workers' rejection of the government's proposal for regulation. Workers' resistance to it or to inclusion in the CLT also reflects their appreciation of autonomy in their work. Although most of them want to expand their rights (Datafolha, 2023; Festi et al., 2024), their refusal of formal salaried work represents a rejection of the subalternity associated with the Fordist labor regime. Although subjected to meticulous algorithmic governance, even perceived as arbitrary in the case of platform layoffs (Seto, 2024), workers, mostly young, prefer this impersonal system of control and the formal freedom to define their working hours to the harassment and violence common to human management (Festi et al., 2024) in a country with a slave tradition. On the other hand, the debate on data transparency, especially in relation to dismissals, is beginning to emerge among workers (Festi et al., 2024), although it is not emphasized in the proposed Brazilian regulation.

From the point of view of political actors, criticism of PL 12/24 comes from both the far right (Brigatti, 2024) and the radical left [Antunes, 2024; Partido Comunista Brasileiro (PCB), 2024; Ribeiro, 2024]. While the far-right attacks the role envisaged for trade unions and claims that regulation will drive platforms out of the country (Moncau, 2024), the left criticism claims that the bill normalizes precariousness, creating a precedent for the end of the minimum wage (Ribeiro, 2024) and labor ties, with the potential generalization of the new figure of the self-employed worker (Antunes, 2024). In addition, left-wing organizations denounce the bill as a copy of proposition 22, presented by Uber in California [cf. (Partido Comunista Brasileiro (PCB), 2024)]. The regulation of platform work proposed by the Lula government means ending any possibility of recognizing that these workers have full rights under general labor legislation. Specific regulation, rather than a mechanism to protect workers, becomes a mechanism of legal certainty for companies by reducing the risks of labor disputes, institutionalizing an inequality between the different sectors of workers.

In the case of Portugal, platformized work has also seen the emergence of new labor actors outside of the classic protagonists. In the case of app-based drivers, the National TVDE Association stands out; in the field of delivery, movements such as “Estafetas em Luta” (created in 2019) or the more recent “Association of Immigrant Workers by App” (born in 2024). On the platform side, a “Portuguese Digital Applications Association” has also been set up. None of these organizations are affiliated to any trade union or employers' confederation.

It should be noted that, in Portugal, the multiplication of precarious work arrangements and the growing de-laborization of a range of economic activities in Portugal (Leite, 2013; Soeiro, 2024a,b), of which platforms are one of the most expressive examples, has gone hand in hand with a progressive blockage of collective bargaining, with falling unionization rates and coverage by up-to-date collective agreements, a phenomenon that is even more pronounced in all the groups furthest from collective voice mechanisms and established forms of worker representation. The number of unionized workers stood at 615,000 in 2019 and the coverage rate of updated collective agreements or new collective agreements was only 30% in 2018, compared to around 60% a decade earlier, and well below the coverage rate of collective agreements in force in both cases (cf. da Paz Campos Lima and Naumann, 2023).

In the case of digital platforms, there is no experience of collective bargaining in this field. Although some hotel and transport unions have advocated extending “pre-digital” collective agreements from the transport and catering sectors to platforms, this has never happened. One “traditional” union stood out for its work with app drivers: STRUP—Sindicato dos Transportes Rodoviários e Urbanos de Portugal, affiliated to the CGTP. This union initially managed to mobilize and organize a working group of digital platform drivers to recognize their rights and improve working conditions, particularly during the pandemic, with the establishment of a 16-point list of demands to be discussed by central government bodies (Costa et al., 2022). In the case of delivery, there have been some contacts between informal movements and the Sindicato de Hotelaria do Norte, but these have not led to the organization of couriers or any collective bargaining process (Costa et al., 2022). Thus, the most recent protests, namely the national demonstrations by passenger transport drivers and the stoppages/strikes by couriers, are not involved from the unions. Their organizers, who gravitate around WhatsApp groups created for the purpose, with the participation of many hundreds of drivers and couriers, organize mainly through social networks (Soeiro, 2024a,b). In the case of TVDE, the aforementioned National TVDE Movement Association makes a point of separating itself from the trade union universe and brings together both workers and owners of intermediary companies. In the case of the delivery sector, the mobilizations have been led by a small group called “Couriers in Struggle.” The main demands of these mobilizations do not include the recognition of subordinate employment contracts. The priorities are above all regulation of the sector, limiting the power of the platforms and improving fares, through: (i) setting absolute values for base fares; (ii) a maximum intermediation fee of the value of the lowest journey; (iii) paying a fee for the route traveled to the customer; (iv) face-to-face exams for access to the sector at the Institute of Mobility; and Transport; (v) greater transparency in the operation of algorithms and the existence of physical headquarters for the companies that own the platforms, especially in the case of delivery.

Digital platforms do not see themselves as employers and are not formally represented by the employers' confederations that are present in institutionalized social dialogue mechanisms, such as the Permanent Social Dialogue Council. However, the Associação Portuguesa das Aplicações Digitais (APAD) and the employers' confederations took similar positions in the process of discussing and approving the new presumption of labor for digital platforms, arguing that excessive regulation should not be imposed on the sector and that workers are autonomous, and their activity should be classified as self-employment. The digital platforms, for their part, have contested the cases in which the courts, in agreement with the Public Prosecutor's Office, have recognized the existence of a subordinate employment relationship between platforms and delivery workers (in the case of drivers, there has never been a court ruling on this aspect).

In the political field, two debates must be distinguished. The first, in 2018, around the so-called “lei Uber,” pitted the more left-wing parties on the one hand, and the government (center-left) and right-wing parties on the other. Thus, the left was against the approval of the law on the grounds that it liberalized a regulated sector (taxis) and did not guarantee workers' rights; the center-left government and the right wanted to legalize Uber's activity, claiming that the new law responded to a legal vacuum (Guerreiro and Marques, 2023). In the debate on the presumption of employment included in the general labor law in 2023, the opposition was between the center-left government and the left, who were both in favor of recognizing the subordinate employment relationship with digital platforms; and the right, who opposed this measure, arguing that it was a legislative rush with potentially harmful economic effects, so much so that it would still be necessary to wait for the European directive on the subject, which at the time was still being debated (Soeiro, 2022). However, one of the debates that also took place in the Portuguese case was about the role of intermediaries, with the center-left government wanting to maintain the figure of the “TVDE operator,” criticized by the left.

As far as the Spanish case is concerned, it is useful to analyze separately the private passenger transport sector and the home-delivery sector.

In the first case, it is necessary to highlight the firm opposition carried out by the different taxi trade associations, especially active in the case of Barcelona and Madrid. Their complaints managed to stop the first introduction of Uber under the collaborative model, as well as taking the issue to the court in Strasbourg, where they obtained a ruling favorable to their interests with implications for the entire European Union. In 2017, the European Court of Justice ruled that Uber's activities should be considered as the provision of a transport service, and not exclusively as an application operating under the legal regime relating to information society services.

As for the role of trade union organizations in this sector, their action can be considered too hesitant, at least initially, with the labor model that was finally implemented, causing confusion and initial disorientation for the large trade union centers (Riesgo Gómez, 2023a). It is only thanks to the action of small groups of workers in coalition with the middle management of the trade union organizations that a common strategy aimed at defending workers' rights was established.

On the part of the political parties, as Guerreiro and Marques (2023) point out, a range of positions can be identified, from the position of the liberal Ciudadanos party, which at all times defended the absolute deregulation of the sector, to the Partido Popular, with contradictory positions within the party, divided between a faction more in favor of protecting the rights acquired by taxi drivers and another close to liberalizing positions. Meanwhile, the further to the left of the ideological spectrum, the stronger the defense of taxi drivers' rights, framed within a discursive framework of opposition to mass uberization and the deregulation of labor relations.

As for trade union membership, it must be said that Spain is characterized by a model that combines low rates of trade union membership with high collective bargaining coverage (Martínez Pastor, 2022). Thus, a large part of the efforts of trade union organizations are focused on obtaining delegates in company-level elections in order to obtain a good position at the collective bargaining tables. These collective agreements, once approved by the trade union and employer sides, come into force and cover all workers in the sector, regardless of whether or not there is trade union representation in each company or whether or not the workers are members of a trade union. Furthermore, the collective agreement can only improve the working conditions laid down in general labor legislation, such as the Workers' Statute or other laws that may be passed in parliament, but never worsen them.

For this reason, the employment relationship through intermediary companies holding VTC authorizations, some directly linked to the platforms (Riesgo Gómez, 2023c), the framework finally established in the private transport sector for the Spanish case, has been the main obstacle to a full platformization of labor relations in this sector. Unlike in most of the places where Uber succeeded in establishing itself, in the Spanish case many platform drivers have minimal protection of their labor rights thanks to collective bargaining.

In the case of food delivery platforms, there was not an interest group with which they were in competition, which allows for further initial growth without stirring up controversy. It is, as mentioned above, thanks to the collective actions of organized groups of workers dissatisfied with their situation that the issue is brought to the public agenda. Thus, the actions of *Riders por Derechos*, a group of workers organized in a horizontal manner at the beginning, managed to gain the support of trade union organizations that are widely established (Fernández-Trujillo Moares and Gil García, 2021; Soto, 2023), establishing solid and stable alliances in terms of advice and legal coverage. Based on this alliance, and the resonance achieved by the protest actions carried out by this grassroots trade union collective, *Riders por Derechos* became a valid interlocutor, recognized by the Minister of Labor of the government herself, holding meetings with them (El Salto, 2020) during the period in which the so-called Ley Rider was forged. This law can be considered as a commitment by the Left coalition government to regulate the sector and recognize the labor rights of platform delivery drivers, being approved in 2021, despite opposition in parliament from parties on the right of the ideological spectrum, both the extreme right embodied in Vox, and the moderate right of Ciudadanos and Partido Popular. As highlights, in addition to the presumption of labor, it is worth noting the obligation of the platforms to allow workers' representatives access to the algorithms responsible for the distribution of the workload, although there is no news of the unions using this resource and, to date, it has had no practical consequences.

## 6 Alternatives to platform capitalism: absences and presences of the cooperative way in the three countries

In addition to the increase in collective organization of workers within and against the platforms, Brazil is a laboratory for self-organization around local platforms that are alternatives to the hegemonic logic of Big Tech (Table 3). In cities with up to 40,000 inhabitants, there are several successful experiments in which drivers are supplanting Uber on the local market using their own platforms (Bonfim, 2024). In the delivery market, Brazilian start-ups like AppJusto build their business model around decent work for workers (AppJusto, 2024), while delivery collectives like Señoritas Courier start from an intersectional perspective on work, self-organizing exclusively women and trans people (Grohmann, 2022).

Not all of these experiences fall under the umbrella of platform cooperativism, constituting a diverse spectrum of workers who don't necessarily organize themselves as cooperatives or develop their own platforms (Grohmann, 2022). For example, the technology center of the Homeless Workers' Movement (MTST), a movement traditionally fighting for housing, developed the chatbot “Contrate quem Luta” (Hire those who Fight), which allows users to hire workers from urban occupations via WhatsApp. The choice of WhatsApp, whose data consumption is free on Brazilian mobile internet services (Seto, 2021), stems from the hardware and network access limitations faced by the majority of Brazilian workers, which make it difficult to adopt new applications.

Another challenge to be overcome is the fragmentation of these local experiences, with the possibility of national and international federations emerging between collectives that share knowledge and technological infrastructure. However, this scenario requires attention to the territorialization of technologies, as in the case of Coopcyle, the European federation of delivery cooperatives, whose initial veto on the use of motorbikes hindered the transfer of its technology to Latin American cooperatives, given that the exclusive use of bicycles was not an immediately viable reality in Latin America (Kasparian, 2022).

In the field of public policies, the manifesto “Action Plan for Platform Cooperativism in Brazil” (2022) and the first meeting “Platform Cooperativism: What Public Policies are Possible?” by the Ministry of Labor set out some possibilities for supporting workers' platforms: tax exemption for companies that hire cooperative platforms; prioritization in state purchases and a reduction in the tax burden for cooperatives; as well as public incubators for cooperative platforms with administrative and accounting advice. The impact of these policies, such as the preference for the services of cooperative and public platforms in state purchases, would not be negligible, given that the Brazilian Federal Government is the country's largest consumer, with annual spending reaching US$23 billion, around 6.7% of GDP (Ribeiro and Inácio, 2019). In addition, public platforms such as TaxiRio, developed by Rio de Janeiro City Hall, already offer viable alternatives to private platforms, with 13.9% of Brazilian platform workers working via taxi apps (IBGE, 2022). TaxiRio has 25,600 drivers (Extra, 2023) and has a daily average of 23,230 journeys in Rio de Janeiro (O Dia, 2020).

In this respect, the Brazilian case is in stark contrast to the Portuguese case. In Portugal, there have so far been no experiences of platform cooperatives in the area of passenger transport or delivery. During the pandemic, Lisbon's city council debated the possibility of creating a public food delivery platform. But this proposal never went ahead. Labor organizations have had a more reconfigurative agenda (demanding greater legal regulation of these economic activities and limiting the power of the platforms) than a prefigurative one (with experiments in self-organization for the provision of this activity or the creation of electronic devices managed by workers).

In Spain, on the other hand, alternative economic forms of platform work have had some space, especially in the delivery sector. In the case of the passenger transport services, due to the high prices that VTC licenses reached on the secondary market that developed after the possibility of obtaining new licenses was closed, alternative solutions of self-organization through the formation of cooperatives are practically unthinkable, as the market is completely under the control of the platforms, with the ability to unilaterally decide essential aspects such as fares or the distribution of the workload.

However, in the bicycle home delivery sector, in addition to the workers' struggles to have their labor rights recognized, which crystallized with the passing of the Rider Law, but did not end there (Diez et al., 2024), for a time cooperative projects aimed at breaking the dominance of the platforms in the sector multiplied. Thus, with direct connections to the Riders for Rights movement, 2018 saw the emergence of two cooperative initiatives with a high symbolic power: La Pájara in Madrid and Mensakas in Barcelona, both integrated in turn into the CoopCycle network, a federation of bicycle delivery worker cooperatives, which brought together more than 60 cooperatives, mostly in Western Europe, and provided its own software with which to operate. While this can be seen as an alternative to the monopolistic dominance of the platforms in the sector, the fact is that competition with the platforms is particularly fierce, challenging the economic viability of this alternative model. In fact, in January 2024, after almost 6 years of activity, the members of La Pájara have decided to close down (Novo, 2024) due to problems related to the financial sustainability of the project.

## 7 Discussion: trends, variations, and similarities between platform capitalisms

Comparing Brazil, Portugal, and Spain reveals several common characteristics of platform capitalism. The platform labor market is strongly oligopolised in all three countries, resulting in a significant dominance of large digital platforms that strongly influence working conditions. However, there is a relevant difference between drivers and couriers: the fact that passenger transport was previously regulated in each country before the entry of platforms led to early pressure for labor regulation for drivers compared to couriers, who emerged in a previously more unregulated market. In this respect, only the Portuguese case goes in the direction of general legislation to bring platform workers, if certain signs are verified, back into the subordinate labor relationship. In the Spanish case, the new riders' law applies to a specific sector. And in the Brazilian case, all the proposed legislation deals with a narrow perspective of platform labor, focusing on drivers and, potentially, delivery workers. However, as we know, platform work is generalizing to different activities: micro tasks, Artificial Intelligence training, care, and communication services (IBGE, 2022; ILO, 2024). Paradoxically, these sectors remain unprotected, while the generalization of the figure of the autonomous platform worker proposed in the new regulation to other activities could mean the intensification of the precariousness of these markets (Ribeiro, 2024). This issue could be tackled in Europe with the new Directive on working conditions on platforms with a view to correctly classifying their professional status and with the debate planned for the ILO international conference in 2025 (ILO, 2024).

In all three countries there is also a racialization of platform work. In Spain and Portugal, there is a strong presence of foreign immigrants in the labor force, while in Brazil Black workers predominate in this market. From the point of view of income, it seems important to mention this particular feature of the platformization of work in Brazil: the average platform worker in this country has a higher income than others, especially in the poorest regions of the country (IBGE, 2022), which poses the challenge of thinking about the specific regulation of this modality in a general scenario of informality. The composition of the labor force in terms of territorial origin is also different in the three countries. In Portugal, immigrants are very important in the passenger transport sector and are the majority in the delivery sector. In Brazil, foreign immigration is not significant, but there are significant regional differences in income between workers from rich and poor regions of the country. The presence of migrant workers on the platforms must be understood in the light of “racialized regimes of accumulation” (Fraser, 2022, p. 41; Fonseca and Soeiro, 2024), which seek to articulate economic exploitation and the political expropriation of workers. Digital platforms battle and aggressive lobbying against regulations is justified in the name of technological innovation and with the idea that the platform would enable populations excluded from the labor market to work. However, the motivation and gain of digital platforms are not moral: they recruit these racialized workers, attract them from other sectors to get a surplus of drivers and couriers in relation to demand, and create a labor pool that lowers prices. In turn, for some immigrants, namely those who have difficulty with the language, platform work becomes a subsistence economic alternative and a way to escape other unskilled jobs, accessing a formal time autonomy they often use to increase their working hours and their level of pay (Bernard, 2023).

The growing organization among platform workers, including racialized workers, but not only, presents challenges common to the cases studied. In all three countries, the difficulty traditional trade union structures are having with this new type of work has led to a new generation of autonomous platform worker organizations, particularly among delivery workers, such as “Estafetas em Luta” in Portugal, “Riders por Derechos” in Spain and the “Aliança Nacional de Entregadores” (ANEA) in Brazil. These organizations have emerged mainly as collectives of workers connected via social networks such as WhatsApp and Telegram. Therefore, in addition to the platformization of work, there is a platformization of workers' sociability, where a sense of community emerges from online relationships that allow for the organization of protests, stoppages, and pickets, which gain territorial expression in symbolic places. In the case of delivery workers, online communities are added to their interactions at meeting points in the vicinity of delivery and bicycle collections, constituting a hybrid public space (Castells, 2015), although even when they are together their attention is mainly directed at smartphones while waiting for service or on social apps.

At the same time, organizations have sprung up in all countries, driven by the digital platforms themselves, which seek to organize and claim to represent workers in line with the interests of companies in the legal debate, arguing that workers are hostile to the existence of formal contracts. In the Portuguese case, the “Movimento dos Estafetas” acts as a proxy for a business organization, in Brazil, Ifood organizes national meetings of delivery workers, while in Spain, similar movements have been observed, such as the Asociación Profesional de Riders Autónomos. Uber's common role in legislative lobbying also highlights the relevance of actors seeking to influence the regulation of platform labor on a global scale, challenging national sovereignties.

The demands of workers' movements in all countries focus mainly on remuneration, such as the demand for an absolute minimum amount per trip and the dispute over the brokerage fee and payment per kilometer. There is a general fatalistic acceptance of algorithmic intermediation, seen as an inescapable mechanism for regulating labor based on supply and demand. However, there is also individualized algorithmic resistance, where workers develop tactics to circumvent the rules of the apps or manipulate the system's variables according to their interests. And although algorithmic work management is widely naturalized, and even in some cases preferred to human management, there is partial questioning, especially in relation to arbitrary dismissals of workers, which occur without the right to defense or appeal.

As well as factors common to all three countries, it is important to highlight similarities that occur bilaterally. Portugal and Spain share several similarities in the regulation of platform labor. Both countries have passed laws that recognize the employment relationship of platform workers, under certain conditions. However, legal formalization presents the challenge of actually implementing and enforcing these laws in the labor market, which still has a strong presence of informal and migrant workers. Regulation without proper enforcement results in a paradoxical situation: while companies can't prevent certain rights from being enshrined formally or performatively (see the principles enshrined in the European directive), workers don't have enough power to ensure that these rights are enforced in real labor relations. Furthermore, both Portugal and Spain, as members of the European Union, are influenced not only by national legislation, but also by the weight of European legislation. This legal and political context contributes to the complexity of regulating platform work, especially with regard to the protection of workers' rights and the inclusion of migrants in the labor force.

Brazil and Spain share the fact that, in addition to the presence of global platforms, they have their own national platforms with significant weight in their local markets and which expand into neighboring countries, in what can be described as “platform sub-imperialism” (Seto, 2024). The centrality of national platforms like iFood in the Brazilian case expresses the country's particular place at the intersection between dependent capitalism and platform capitalism (Seto, 2024) and how the political-economic history of each nation influences the regulation of labor. In contrast to the hostility of the Big Techs of the Global North to unionization (Felitti, 2022), iFood has directly organized workers' forums and academic and third sector initiatives (Grohmann, 2022), seeking consensus among the other actors around the legal regulation of platform work rather than coercion.

Another similarity between Brazil and Spain is the judicialization of platform labor relations, while in Portugal court cases are very recent, also due to the creation of the unique figure of the TVDE operator (which legally eliminated any possibility of the platform being held liable for employment) and the fragile citizenship status of migrant workers in the delivery sector.

Brazil and Spain also have experiences of co-operative platforms. In Latin America, there are examples such as Señoritas Courier and public taxi platforms, with significant market dominance, and in Spain, the La Pájara and Mensakas co-operatives, which are part of the CoopCycle network (Grohmann, 2022). However, the economic viability of these alternative models depends on public policies that encourage the co-operative sector.

Brazil and Portugal, on the other hand, share similarities, especially in terms of workers' demands and the platform companies' strategies for winning over public opinion. In both countries, the main demands of the collective mobilizations among delivery workers do not focus on the recognition of employment, but rather on an immediate economic programme. And in these countries, the platform companies have also promoted opinion polls among their workers to influence the public debate that the categories do not want labor regulation and recognition, co-opting intellectuals and research institutes to legitimize this work. Another common point is the emergence of new employers' associations in both countries to represent the interests of the platforms.

Finally, in order to understand the process of regionalization of the platformization of work, it is important to summarize the specificities of each country. In Spain, the history of broad coverage of labor legislation and collective bargaining favored the early regulation of drivers' work and, along with Riders' struggles for rights, the recognition of employment via a Supreme Court ruling even before the Rider law, which also guaranteed principles of algorithmic transparency for workers. The Spanish scenario is also unique for the relationship of solid alliances between traditional trade unions and the new collectives of platform workers. In contrast, the judges of the Brazilian Supreme Court decided contrary to that of Spain, in a context of structural labor informality aggravated by the Temer government's neoliberal labor reform and workers' hostility to official trade unionism.

While in Europe there seems to be a “predatory inclusion” (McMillan Cottom, 2020) through platform work, with the inclusion in the labor market of segments that are more exploited due to the greater vulnerability of their citizenship status, in Brazil platform work is expanding in a context of generalized precariousness in labor relations due to its character as a dependent economy. In this sense, platform capitalism expresses the legacies of the colonial processes in each society and, at the same time, the contemporary role of national capitalism in each country. This is why Portugal is completely dominated by foreign platforms, unlike Brazil and Spain, where locally-based platforms compete with large transnational companies.

## 8 Conclusion and future research

The comparison between Brazil, Portugal, and Spain highlights how different national contexts regionally shape the global phenomenon of platform capitalism. The similarities observed, such as the monopolization of markets and workers' resistance, show that the challenges faced are common, despite local specificities. Workers' organization reveal a global movement in search of better working conditions and recognition. It is important to note, in this context, the difficulties faced by traditional trade unions and the challenges faced by new types of platform workers' organizations. At the same time, the initiatives taken by digital platforms to create forms of worker representation and organization highlight the need for a critical analysis of these processes and for attention to be paid to regulatory processes, to the weight of corporate lobbying and the cultural battle (Diez et al., 2024) driven by platforms, aimed at denying the application of labor law, the defense of the right to free business establishment, including in their conception of business a part of their own employees considered as autonomous workers.

Investigating how different national social formations affect the regulation of digital platforms provides valuable insights for the formulation of public policies. We can see how the regulation of work on platforms varies significantly, influenced by trade union traditions, each country's labor legislation, and demographic and legal factors unique to each country. For example, the strong coverage of collective bargaining among Spanish workers contrasts with the structural informality of Brazilian labor, and in Portugal, the creation of the TVDE regime has discouraged lawsuits compared to those in the Spanish and Brazilian courts. Understanding the interaction between the global nature of the platforms and the national contexts is essential in order to identify and combat inequalities specific to each context, as well as possible global alliances for decent work.

The workers' struggle, despite the emergence of common characteristics, is also diverse. In Spain, the relationship between unions and new associations stands out, in Brazil platform cooperativism and incipient public platforms, while in Portugal immigration is a decisive element in the organization of relays. It is worth mentioning the strong racialization of platform work in all three countries. From the point of view of labor protection, the level of social protection in European countries, despite the challenge of implementing legal provisions, is still higher than in Brazil. Being a relatively fragile economy in the south of Europe offers better working conditions compared to being a leader in the global south.

But the particular role of each country in the global economy also influences the operation of the platforms. The insertion of Brazil and Spain in platform capitalism demonstrates their superior relevance to the Portuguese economy, evidenced by the development of their own platforms that combine the ability to attract foreign capital with national capital and research and development resources. This combination integrates the technological and financial dimensions of platform capitalism, reinforcing the importance of these countries on the regional stage. A hypothesis to be developed in future work is the constitution of regional platform sub-centers in geopolitics and the regionalization of the platformization of labor. Although they don't compete on a global scale with North American and Chinese platforms, these platforms extend their dominance over neighboring countries, such as the Spanish platforms in Portugal, standing out within their respective regions.

In addition to the on-location platforms being officially considered employers, there is a broader debate about the labor framework and social protection of the group of professionals who are dependent on platforms. For example, content creators on social networks are subject to the governance of these platforms, with changes to recommendation systems and revenue transfer formulas, affecting entire entertainment and journalism production chains. In addition to regulating labor relations, it is important to debate the regulation of the monopolistic nature of digital markets, given that cooperative platforms or those that seek to offer decent working conditions suffer from powerful barriers to entry. The role of the state goes beyond regulating working conditions, because whether through public platforms or encouraging cooperative platforms, intensifying the entry of these platforms also increases the relative power of workers.

Finally, it seems necessary to consider the importance of the control of the data that the large technological platforms operating on the ground can accumulate, as well as their role in influencing the regulatory attempts made by public policy makers. Ultimately, aspects such as the length of the working day, the income earned by drivers and delivery workers, or even the real possibilities of articulating resistance processes that transcend the offline world, are strongly influenced by the asymmetrical handling of information by the platforms and their possible instrumental use. Although it is not an aspect that we have been able to develop in this article, the possibility of introducing regulatory mechanisms aimed at increasing transparency and traceability of the algorithms that govern the work process could have effects in the sense of significantly improving the working conditions of these workers. This is also where the debate on the role of public platforms acting as repositories of last resort for data collected by private companies in real time, or as mandatory gateways for service requests, can be situated.

## Data Availability

The original contributions presented in the study are included in the article/supplementary material, further inquiries can be directed to the corresponding author.
